# Development of a natural language processing pipeline for assessment of cardiovascular risk in myeloproliferative neoplasms

**DOI:** 10.1002/hem3.143

**Published:** 2024-08-08

**Authors:** Andrea Duminuco, Joshua Au Yeung, Raj Vaghela, Sukhraj Virdee, Claire Woodley, Susan Asirvatham, Natalia Curto‐Garcia, Priya Sriskandarajah, Jennifer O'Sullivan, Hugues de Lavallade, Deepti Radia, Shahram Kordasti, Giuseppe Palumbo, Claire Harrison, Patrick Harrington

**Affiliations:** ^1^ Department of Haematology Guy's and St Thomas' NHS Foundation Trust London UK; ^2^ Haematology Unit with BMT A.O.U. Policlinico “G. Rodolico‐San Marco” Catania Italy; ^3^ Cogstack, Guy's and St Thomas' NHS Foundation Trust London UK; ^4^ School of Cancer and Pharmaceutical Science, King's College London London UK

A central feature of myeloproliferative neoplasms (MPN) is an increased risk of cardiovascular thrombotic complications, and this is the primary determinant for the introduction of cytoreductive therapy.^1^ The landmark ECLAP study in polycythemia vera (PV) patients, showed cardiovascular mortality accounted for 45% of all deaths, with a thrombosis incidence rate of 1.7/100 person/year and a cumulative incidence of 4.5% over a median follow‐up of 2.8 years.^2^

Natural language processing (NLP) is a branch of machine learning involving computational interpretation and analysis of human language. CogStack (https://github.com/CogStack), is an open‐source software ecosystem, that retrieves structured and unstructured components of electronic health records (EHR). The Medical Concept Annotation Toolkit (MedCAT), the NLP component of CogStack, structures clinical free text by disambiguating and capturing synonyms, acronyms, and contextual details, such as negation, subject, and grammatical tense, and mapping text to medical Systematized Nomenclature of Medicine–Clinical Terms (SNOMED‐CT) concepts. This technique is known as “named entity recognition and linkage” (NER+L). MedCAT has previously been used and validated in many studies to structure EHR data across a range of medical specialties for auditing, observational studies, de‐identifying patient records, operational insights, disease modeling, and prediction.^3^, ^4^, ^5^, ^6^, ^7^, ^8^

We employed our NLP pipeline, Cogstack, and MedCAT, to determine the prevalence and impact of cardiovascular risk factors upon thrombotic events during follow‐up. We used Cogstack to retrieve outpatient hematology clinic letters and hematology discharge letters. MedCAT was then used for NER+L of relevant clinical free‐text to respective SNOMED‐CT codes that were determined by two hematology specialists. The base MedCAT model was trained unsupervised on >18 million EHR documents, and this was further fine‐tuned using a 80:20 train:test split with 600 clinician‐annotated MPN‐specific documents. Total SNOMED‐CT code counts were aggregated and grouped by individual patient, a unique threshold count was then applied to “infer” presence of the respective SNOMED code. In this process, hematology specialists read through clinical documents and manually highlight correct words or phrases detected by MedCAT that correspond to the SNOMED concept of interest.

We deploy a two‐step validation process that has been well described.^3^, ^8^ The first is to evaluate and validate the NER model performance on a document level demonstrating how accurately MedCAT is able to identify the medical concepts of interest. This involves hematology specialists annotating medical concepts and comparing this to the model NER outputs (Supporting Information S1: Table 1). The second step involves manual validation by creating a gold‐standard real‐world dataset. Two hematology specialists were randomly assigned to review a subsample of the patient sample's clinical notes (*n* = 112 [20%] in ET cohort, and *n* = 60 [17%] in the PV cohort) and, taking into account the entire clinical history, state whether the patient had presence or absence of the selected SNOMED concepts (Supporting Information S1: Tables 2 and 3). Finally, using the manual‐validated data set, a threshold optimizer was used to find optimal concept counts for real‐world F1 inference. The manual validation is crucial because whilst ML models can mislabel concepts on a document level, the key factor is how well a model performs in the real‐world at recognizing the presence or absence of a clinical entity on a patient level.

The selected SNOMED concepts were cardiovascular risk factors, including hypertension (HTN), hypercholesterolemia (HC), diabetes mellitus (DM), smoker status, and obesity. We also assessed for cardiovascular events, including portal vein thrombosis (PVT), deep vein thrombosis (DVT), pulmonary embolism (PE), myocardial infarction (MI), stroke/cerebrovascular accident (CVA), cerebral sinus thrombosis (CST), and thrombosis not otherwise specified (NOS). A schematic representation of the process is represented in Figure 1A.

**Figure 1:**
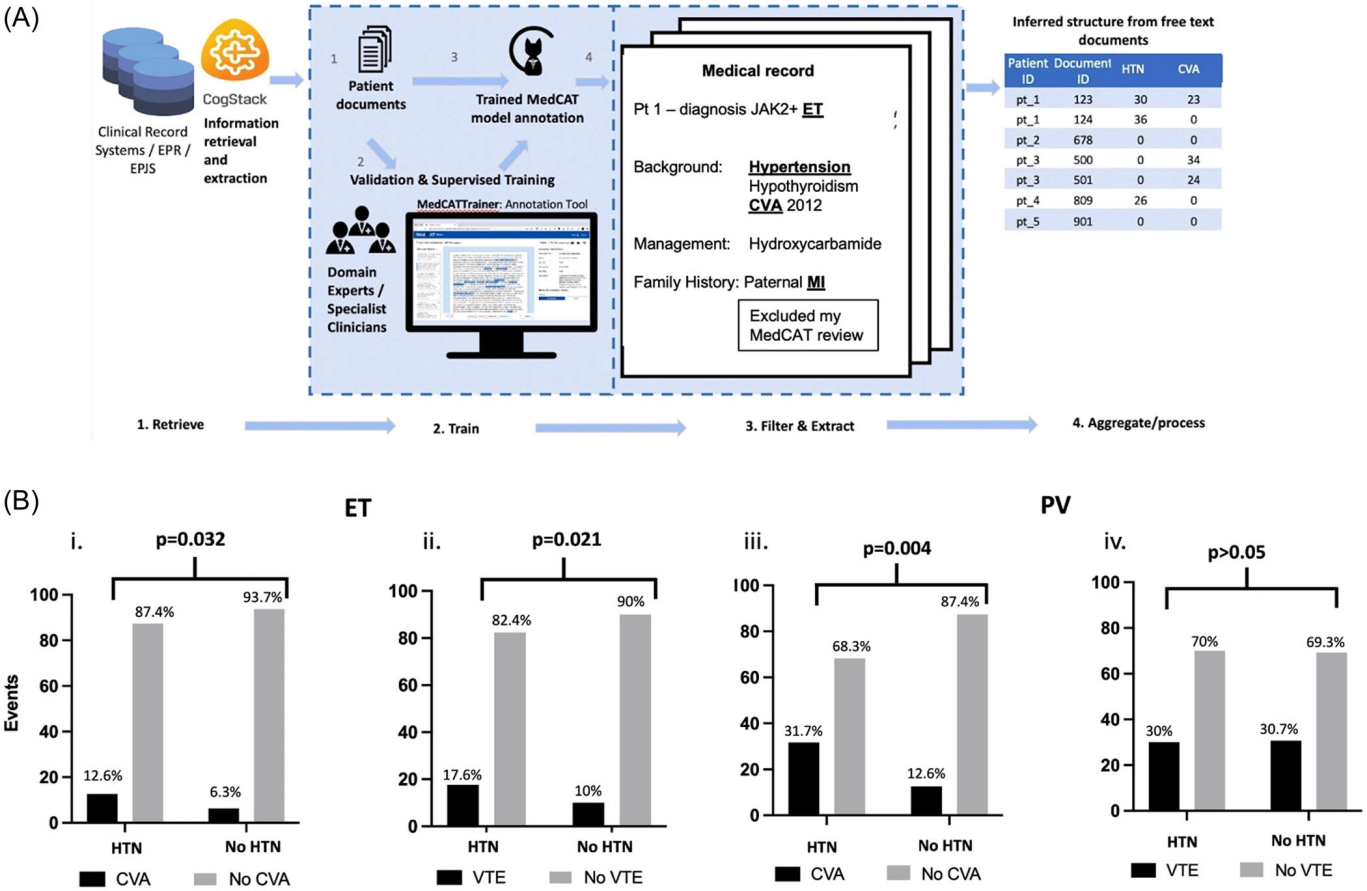
(A) Data from specific myeloproliferative neoplasms (MPN) patient documents is extracted from electronic health records by Cogstack. The MedCAT model is trained using MPN‐specific documents and then can be used to interpret and map text to SNOMED‐CT concepts, with the total number aggregated and grouped by individual patient. Manual validations were performed to confirm accuracy. (B) Relationship between cardiovascular risk factor and type of thrombotic event, with a statistically significant correlation between hypertensive disorder (HTN) and cerebrovascular accident, in both ET (i) and PV (iii) cohorts, calculated using Fisher's exact test. Increased incidence of venous thromboembolism in HTN patients also observed in ET cohort (ii) but statistical significance not reaches for HTN in PV cohort (iv).

Data from 360 PV and 560 ET patients, reviewed at Guys' and St Thomas NHS Foundation Trust (GSTT) for at least one visit between January 2005 and April 2023, were evaluated (Supporting Information S1: Table 4). A total of 12905 documents from 560 ET patients (median 20 per patient, interquartile range [IQR], 8–34), and 11250 from 360 PV patients, (median 27 per patient, IQR, 11–47), were reviewed. In the manual validation data set (*n* = 112 patients for ET and *n* = 60 for PV), MedCAT achieved excellent real‐world F1 scores with regards to specificity and sensitivity (Supporting Information S1: Tables 2 and 3).

For the ET cohort, using a threshold of >2 mentions to define a positive population, HTN was identified in 21.3% (119) of patients, DM in 4.6% (26), MI in 3.6% (20), CVA in 7.7% (43), NOS thrombosis in 8% (45), DVT in 1.4% (8), PE in 1.8% (10), PVT in 1.3% (7) and positive smoking status in 6.6% (37) (Supporting Information S1: Figure 1A,B). HC was identified in 9.6% (54) using a threshold >1. 52% (56) of patients with HC and 69.2% (18) of those with DM also had HTN. Obesity was not identified in any patients using this approach. Considering overall venous thromboembolic (VTE) occurrence, 11.6% (65) of patients reported an event. Of patients with CVA/MI, 58.1% (43)/55% (11) had this event pre‐ or at diagnosis, and 30.2% (13)/10% (2) while receiving cytoreductive therapy.

For the PV cohort, using a threshold of >2 mentions to define the presence of the condition, HTN was identified in 23.1% (83) of cases, DM in 5.6% (20), MI in 3.1% (11), NOS thrombosis in 19.4% (70), DVT in 2.8% (10), PE in 2.8% (10), and PVT in 5% (18) with only one case of cerebral venous sinus thrombosis identified (0.3%) (Supporting Information S1: Figure 2A,B). Positive smoking habit was reported in 5.6% (20) of patients. Overall VTE was reported in 23.3% (84) of cases. Using a threshold of 1 mention, HC and CVA were detected in 7.5% (27) and 14.2% (51) patients, respectively. In keeping with the ET cohort, obesity was not identified in any patients. CVA/MI events occurred in 58.8% (30)/54.5 (6) cases prior to or at diagnosis and 23.5% (12)/9.1 (1) during cytoreductive treatment. Patients affected by DM suffered from HTN in 75% of cases (15), 66.7% (18) of HC patients had concomitant diagnosis of HTN, while 1.7% of the total had HTN, HC, and DM together.

TE were significantly higher in PV patients in the case of CVA (*p* = 0.002), PVT (*p* < 0.001), venous thromboembolism (VTE, referring to any form of venous thrombotic event in an anatomical region that is not otherwise mentioned, i.e., axillary or retinal veins, *p* < 0.001) and the overall number of TE episodes, was also significantly higher in PV patients than in the ET cohort (*p* < 0.001), as reported in Table 1 below.

**Table 1:** Occurrence of varying types of thrombotic events in ET and PV patients.

Type of thrombosis	ET (560 patients) *n* (%)	PV(360 patients) *n* (%)	*p*
Deep venous thrombosis	8 (1.4)	10 (2.8)	0.15
Pulmonary embolism	10 (1.8)	10 (2.8)	0.314
Myocardial infarction	20 (3.6)	11 (3.1)	0.673
Cerebrovascular accident	43 (7.7)	51 (14.2)	0.002
Portal vein thrombosis	7 (1.3)	18 (5)	<0.001
Cerebral venous thrombosis	6 (1.1)	1 (0.3)	0.177
Thrombosis, NOS	45 (8)	70 (19.4)	<0.001
Venous thromboembolism	65 (11.6)	84 (23.3)	<0.001
Overall thrombotic events	112 (20)	126 (35)	<0.001

*Note*: Venous thromboembolism: any additional form of venous thrombotic event in an anatomical region not otherwise mentioned.

Abbreviation: NOS, not otherwise specified.

ET patients diagnosed with HTN, were more likely to have CVA than those without (of 119 HTN‐affected, 15 had CVA, and of 441 HTN‐negative, 28 had CVA, *p* = 0.032, Figure 1B, i) Patients with HTN were also more likely to have a venous thrombotic episode (of 119 HTN‐affected, 21 had VTE, and of 441 HTN‐negative, 44 had VTE, *p* = 0.021, Figure 1B, ii). Similarly, among PV patients who experienced a CVA, 39% (20) had HTN, demonstrating that, as would be expected, hypertension predisposes to CVA (20:63 vs. 31:246, *p* = 0.004, Figure 1B, iii). However, unlike ET, no correlation was reported between HTN and VTE (19:64 vs. 65:212, *p* > 0.05, Figure 1B, iv).

Considering overall TE, for ET, 31.9% (38) of patients were affected by HTN, 22.2% (12) by HC, 19.2% (5) by DM, and 24.3% (9) had smoking habit reported a TE in their clinical history. Multivariate analysis of the ET cohort (considering HTN, HC, DM, and smoking habit) confirms the central role of HTN in increasing the risk of TE (OR: 2.5; 95% CI: 1.5–4.2; *p* < 0.001). Regarding the PV cohort, 126 (35%) patients had a TE, and 44.6% (37) of HTN‐affected patients experienced thrombosis. 48.1% (13) of HC, 35% (7) of DM, and 25% (5) of smoking habit patients experienced a TE. Applying a multivariate analysis (with the same cardiovascular risk factors described for ET), patients affected by HTN in the PV cohort show a higher risk of experiencing TE (OR: 1.5; 95% CI: 1.1–2.8; *p* < 0.016).

To our knowledge, this is the first time a machine learning approach has been utilized to process and analyse large volume data in the ET and PV patient population, to provide valuable clinical insights. We describe a novel approach to cardiovascular risk assessment in patients with ET and PV, by using our NLP approach, we were able to analyse over 23,000 hematology documents that would have otherwise taken significant human time and labor. The advantage of our approach is that it can give near real‐time updates of clinical events, which can inform patient management and risk prediction. Our manual validation demonstrated adequate performance in identifying a range of cardiovascular comorbidities and TE using this approach.

A previous report of 891 ET patients showed a prevalence of 5% for CVA, 2% for MI, and 4% for VTE, suggesting that our approach's detection rate is within acceptable limits.^9^ Moreover, in an extensive study of Italian hematology centers, 235 patients with PV and 259 with ET were retrospectively evaluated, with a reported occurrence of thrombosis in 20.4% and 13%, respectively. Concerning CV risk factors, smoking was reported in 14.3%, HTN in 46.5%, HC in 12.5%, and DM in 8.2%, with results from these patient cohorts again consistent with our findings.^10^

In keeping with previous studies our data suggest a higher risk for thrombotic events in PV, specifically regarding CVA, PVT, and VTE, when compared with the ET cohort. There was also a greater occurrence of overall thrombosis in the PV patient group. This is likely to partially reflect the greater frequency and higher variant allele frequency (VAF) of JAK2 mutation observed in PV patients. These data provide a basis for further mechanistic analysis to better define differences in thrombotic risk and assessment of the impact of driver mutations within disease groups is also warranted.

We provide a rare “real‐world” report on the prevalence of comorbidities in this patient group. We have shown that hypertension is a comorbidity of great significance with regards to impact on thrombotic risk in MPN, with significantly increased risk of CVA in both PV and ET cohorts. This suggests a particular focus on controlling HTN is warranted and also highlights the need for health education for MPN patients to prevent the onset of hypertension. However, we were unable to assess differences between well‐controlled and refractory or untreated hypertension using this approach. Our data support the routine assessment of cardiovascular comorbidities in defining the thrombotic risk in MPN patients, through the use of novel scoring systems such as QRISK‐3. Studies should explore incorporating these comorbidities into predictive models to stratify patients based on thrombotic risk, as well as assess impact of targeted therapies on reducing associated risk.^11^

A limitation of the NLP approach is that we have prespecified variables of interest for the model to detect, which prevents assessment of the impact of other associated factors, without development of an increasingly complex model. For example, it was not possible to assess the impact of driver mutation status on thrombotic risk using this approach in the current analysis.

Medical language is often complex, and shorthand or abbreviations used may be both department and region‐dependent. For example a doctor documenting “AF” for atrial fibrillation could also mean “artificial feed” when used by dieticians. Using clinician‐annotated documents, we are able to fine‐tune the model to account for these variations. However, the performance of this approach is largely dependent on the quantity and the quality of training data and clinician annotations. Where there is large variation in the spelling, typing errors, context, or syntax, this will usually require more detailed annotations to perform well. This phenomenon is reflected in the somewhat moderate performance of “Thrombosis, not otherwise specified”. We used a broader SNOMED concept to capture thromboses that did not fit into our prespecified feature selection, but for this concept alone there were over 20 word/phrase annotation variations. The model performance could be improved to learn and generalize in broad concepts, but it will require further training over a larger number and range of documents.

Another limitation to the NLP approach is that it fails to capture conditions or statuses that are fluctuant or temporal, or those that are not explicitly stated. For example, for the concept of “Smoker,” MedCAT achieved a document‐level F1 score of 0.87 (see Supporting Information S1: Table 1), however only achieved an F1 score of 0.64 and 0.66 in the ET and PV validation cohorts, respectively. This is because in a series of visits, a patient may well have stopped or restarted smoking, and smoking status may not be explicitly stated, for example, we do not always document that the patient is a “smoker,” instead a clinician may write “the patient smokes 20 cigarettes a day,” or “the patient only smokes socially.”

Finally, like all electronic health record research, the quality of NLP outputs is limited by the data quality of documented free text. For example, there was only one clinician annotation of “obesity” (see Supporting Information S1: Table 1), this would explain the lack of any cases of obesity being detected and suggests this co‐morbidity may not be frequently documented, or alternatively this information may be coded in structured data (i.e., recorded weight and height measurements).

In addition, our study could be limited by the inclusion of analysis of patients with only one visit to our clinic. By analyzing these cases separately and validating them manually, we confirmed that the specificity and sensitivity of the natural processing language model are acceptable for ET and PV. Further studies are required to analyze the use of our NLP pipeline prospectively to define cardiovascular risk in MPN patients.

## AUTHOR CONTRIBUTIONS

All authors have contributed substantially to the following. Andrea Duminuco, Joshua Au Yeung, Claire Harrison, and Patrick Harrington interpreted and analyzed the data and drafted the article: Raj Vaghela, Sukhraj Virdee, Claire Woodley, Susan Asirvatham, Natalia Curto‐Garcia, Priya Sriskandarajah, Jennifer O'Sullivan, Hugues de Lavallade, Deepti Radia, Shahram Kordasti, and Giuseppe Palumbo revised the manuscript for intellectual content: All authors contributed to the article and approved the submitted version.

## CONFLICT OF INTEREST STATEMENT

Hugues de Lavallade has received research grants and honoraria from Incyte and honoraria from Novartis and Pfizer. Claire Harrison has received speaker fees from Novartis, Jannsen, CTI, Celgene, Medscape and has served on the Advisory Board for Incyte, CTI, Sierra Oncology, Novartis, Celgene, Roche, AOP pharma, Geron and Astra Zenica, and is an Editor of HemaSphere. Patrick Harrington has received research funding from GSK, BMS, Novartis, Incyte, AOP, and Constellation and honoraria from GSK, Incyte, and Novartis.

## FUNDING

This research received no funding.

## Supporting information

Supplementary information.

## Data Availability

The data supporting this study's findings are available upon documented request.
