# Supplementary material for: Development of a natural language processing pipeline for assessment of cardiovascular risk in myeloproliferative neoplasms
Source: Hemasphere. 2024 Aug 8;8(8):e143. doi: 10.1002/hem3.143 (PMC11310405; doi:10.1002/hem3.143)
Supplement: Supplementary file 1 — Supplementary information. [file HEM3-8-e143-s001.docx]

**Cogstack Manuscript Supplementary Material**

Supplementary Methods

**Statistical analysis:**

Qualitative results were summarized in counts and percentages.
To evaluate the thrombotic risk and differences between ET and PV, patients with venous clotting events (portal vein, cerebral venous sinus, deep vein thrombosis, pulmonary embolism, and thrombosis not otherwise specified) have been initially grouped, and, to perform overall analyses, all subjects who had at least one thrombotic episode have been considered together.

The frequency of event occurrences linked to comorbidities and the differences between ET and PV cohorts were studied employing the Mann-Whitney U test or Kruskal-Wallis test for continuous data and the chi-squared test or Fisher’s exact test for categorical data.

statistician calculations were performed using MedCalc Statistical Software version 19.2.6 (MedCalc Software bv, Ostend, Belgium; https://www.medcalc.org; 2020) and Prism 9 (version 9.5.1, 528, January 24, 2023).

Model accuracy is presented as Precision, Recall and F1 score which is a metric used to assess the overall performance of a binary classification machine learning model. It offers a balanced measure of a model’s effectiveness in correctly identifying positive instances (true positives) while minimising the impact of false positives and false negatives. The F1 score is calculated as the harmonic mean of precision (ratio of true positive predictions to the total number of positive predictions: the accuracy of positive predictions) and recall (or sensitivity, the proportion of true positive predictions to the total number of actual positive instances: model’s ability to capture all positive instances). It ranges from 0-1, and a higher F1 score denotes a better-quality classifier. Here we present both the performance of the model on a document-level and on a patient-level (aggregation of all selected documents for each individual patient).

Supplementary Results:

Supplementary Table 1

| **cui** | **SNOMED Concept name** | **Concept Count*** | **Precision** | **Recall** | **F1-Score** |
| --- | --- | --- | --- | --- | --- |
| 38341003 | Hypertensive disorder, systemic arterial | 21 | 0.91 | 0.95 | 0.93 |
| 230690007 | Cerebrovascular accident | 9 | 0.69 | 1 | 0.82 |
| 439127006 | Thrombosis | 6 | 0.63 | 0.83 | 0.71 |
| 128053003 | Deep venous thrombosis | 3 | 1 | 1 | 1 |
| 13644009 | Hypercholesterolemia | 3 | 1 | 1 | 1 |
| 17920008 | Portal vein thrombosis | 3 | 1 | 1 | 1 |
| 22298006 | Myocardial infarction | 3 | 0.75 | 1 | 0.86 |
| 59282003 | Pulmonary embolism | 3 | 0.75 | 1 | 0.86 |
| 77176002 | Smoker | 3 | 1 | 1 | 1 |
| 850981000000101 | Cholesterol level | 3 | 1 | 1 | 1 |

Table showing performance (Precision, Recall and F1-Score) of MedCAT (Medical Concept Annotation Tool) on the task of named-entity recognition in a test set of 100 Clinician-annotated haematology documents.

*Concept count denotes how many instances of each SNOMED concept was annotated in total by the clinicians in the test set.

Supplementary Table 2

| **Condition in ET patients** | **F1 Score** | **Precision** | **Recall** | **Disease Count** |
| --- | --- | --- | --- | --- |
| Hypertensive disorder (HTN) | 0.91 | 0.91 | 0.92 | 34 |
| Hypercholesterolemia (HC) | 0.82 | 0.85 | 0.79 | 14 |
| Diabetes mellitus (DM) | 1 | 1 | 1 | 6 |
| Smoker | 0.65 | 0.61 | 0.69 | 16 |
| Obesity | 0.02 | 0.01 | 1 | 1 |
| Deep venous thrombosis (DVT) | 1 | 1 | 1 | 2 |
| Pulmonary embolism (PE) | 1 | 1 | 1 | 2 |
| Myocardial infarction (MI) | 0.67 | 0.67 | 0.67 | 6 |
| Cerebrovascular accident (CVA) | 0.87 | 0.91 | 0.33 | 12 |
| Portal vein thrombosis (PVT) | 1 | 1 | 1 | 2 |
| Cerebral venous sinus thrombosis | 0.86 | 0.75 | 1 | 3 |
| Thrombosis, NOS | 0.73 | 1 | 0.57 | 7 |

A table showing real-world performance (Precision, Recall, F1-score) and disease count calculated for every condition in ET on a patient level, based on the clinician manual validation results (112 patients). ET: essential thrombocythaemia; NOS: not otherwise specified

Supplementary Table 3

| **Condition in PV patients** | **F1 Score** | **Precision** | **Recall** | **Disease Count** |
| --- | --- | --- | --- | --- |
| Hypertensive disorder (HTN) | 0.81 | 1 | 0.68 | 19 |
| Hypercholesterolemia (HC) | 0.92 | 1 | 0.86 | 7 |
| Diabetes mellitus (DM) | 1 | 1 | 1 | 2 |
| Smoker | 0.67 | 0.71 | 0.62 | 8 |
| Obesity | 0 | 0 | 0 | 0 |
| Deep venous thrombosis (DVT) | 0.80 | 0.67 | 1 | 2 |
| Pulmonary embolism (PE) | 1 | 1 | 1 | 1 |
| Myocardial infarction (MI) | 1 | 1 | 1 | 4 |
| Cerebrovascular accident (CVA) | 0.95 | 0.91 | 1 | 10 |
| Portal vein thrombosis (PVT) | 1 | 1 | 1 | 4 |
| Cerebral venous sinus thrombosis | 1 | 1 | 1 | 1 |
| Thrombosis, NOS | 0.69 | 0.54 | 0.93 | 14 |

Table showing real-world performance (Precision, Recall, F1-score) and disease count calculated for every condition in PV on a patient level, based on the clinician manual validation results (60 patients). PV: polycythaemia vera; NOS: not otherwise specified

Supplementary Table 4.

| **Demographics** | **ET**  **(560 patients)** | **PV**  **(360 patients)** |
| --- | --- | --- |
| **Age, n (SD)** | | |
| Mean | 48.9 (+/-16.1) | 50.4 (+/-14.4) |
| **Gender , n (%)** | | |
| Female | 398 (71.1%) | 190 (52.8%) |
| Male | 162 (28.9%) | 170 (47.2%) |
| **Ethnicity, n (%)** | | |
| White: British, English, Irish | 218 (38.9%) | 157 (43.6%) |
| Not Specified / Not Stated / None | 216 (38.6%) | 125 (34.7%) |
| White: Any other White background / Other European | 53(9.5%) | 27 (7.5%) |
| Black or Black British | 29 (5.2%) | 20 (5.6%) |
| Mixed: White and Asian / Black / Other | 10 (1.8%) | 8 (2.2%) |
| Other Ethnic Groups | 22 (3.9%) | 8 (2.2%) |
| Asian or Asian British | 12 (2.1%) | 6 (1.7%) |

Demographics of ET and PV patients

Supplementary Figures:

Supplementary Figure 1


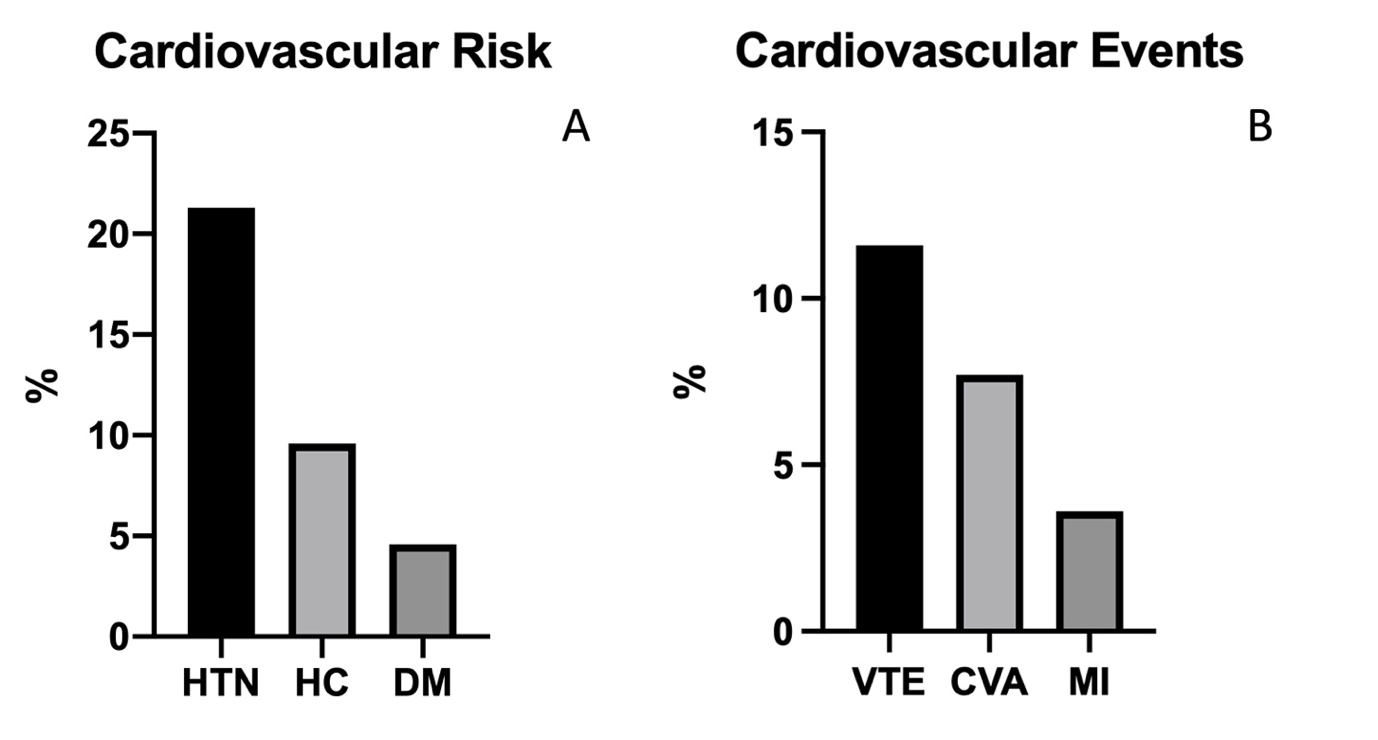


Supplementary Figure 1. Frequency of cardiovascular risk factors (A) and thrombotic events (B) in the ET cohort. HTN: hypertensive disorder; HC: hypercholesterolaemia; DM: diabetes mellitus; VTE: venous thromboembolism; CVA: cerebrovascular accident; MI: myocardial infarction

Supplementary Figure 2.


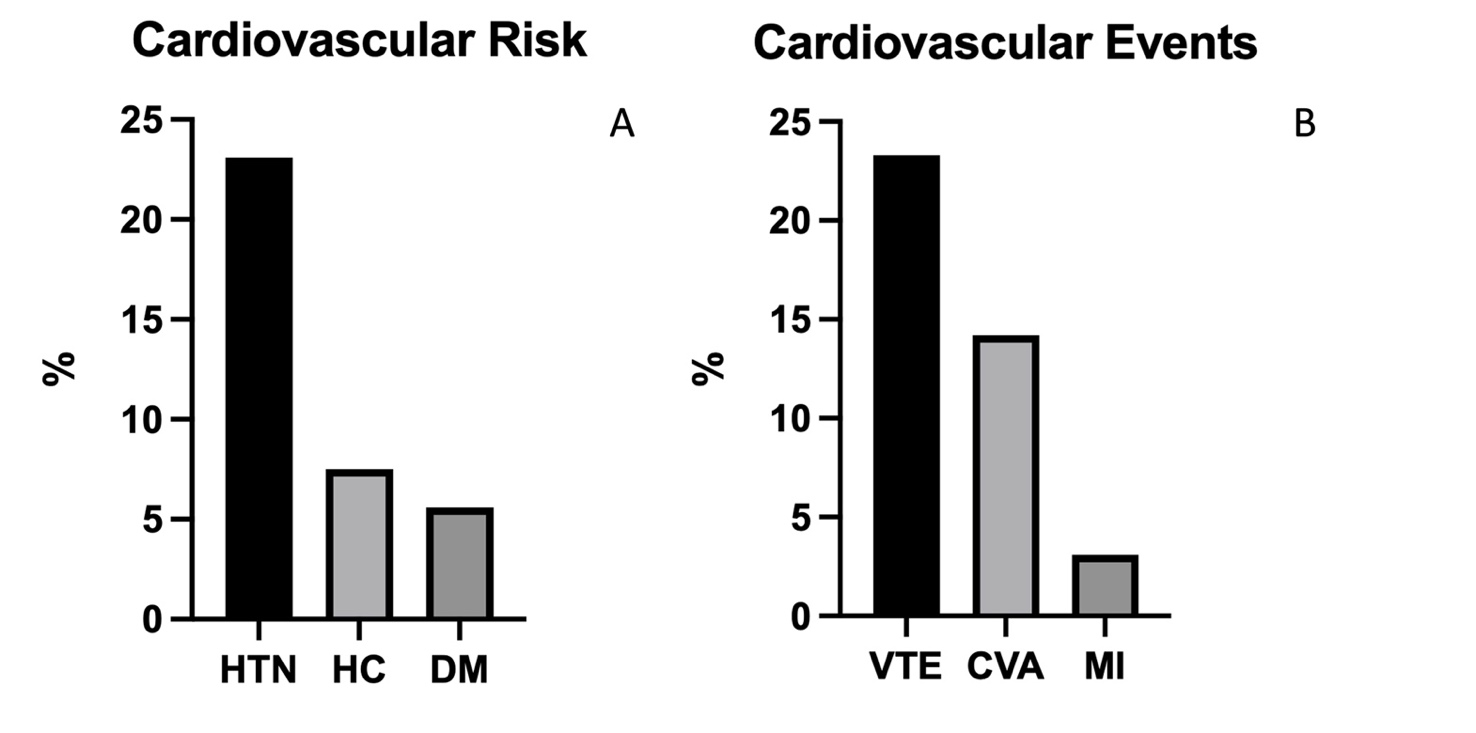


*Supplementary Figure 2. Frequency of cardiovascular risk factors (A) and thrombotic events (B) in the PV cohort. HTN: hypertensive disorder; HC: hypercholesterolaemia; DM: diabetes mellitus; VTE: venous thromboembolism; CVA: cerebrovascular accident; MI: myocardial infarction*
